# A Systems-Based Computational Model of Alcohol’s Toxic Effects on Brain Development

**Published:** 2008

**Authors:** Julia M. Gohlke, Susanne Hiller-Sturmhöfel, Elaine M. Faustman

**Keywords:** Maternal alcohol exposure, prenatal alcohol exposure, fetal alcohol effects, fetal alcohol syndrome (FAS), alcohol-related neurodevelopmental disorder (ARND), neocortex, neurogenesis, synaptogenesis, apoptosis, computational model, animal model, animal studies, human studies, systems biology

## Abstract

Important stages during neurodevelopment include the generation of new nerve cells (i.e., neurogenesis), differentiation and migration of these cells to their final location in the brain, formation of connections with neighboring cells (i.e., synaptogenesis), and cell death of neurons that fail to form the appropriate connections. Research found that alcohol exposure during fetal development can interfere with all of these processes. A systems biology approach using computational models of brain development in different species has been used to determine the relative contributions of alcohol-induced impairment of neurogenesis and synaptogenesis to alcohol-related neurodevelopmental deficits in mice, rats, rhesus monkeys, and humans. The results obtained with these models suggest that alcohol’s impact on cell division during neurogenesis results in greater deficits in neuron numbers in the adult than the alcohol-induced increase in cell death during synaptogenesis. In primates, the accelerated development of susceptible brain regions may convey increased sensitivity to alcohol-induced neurodevelopmental deficits. Systems-based approaches, such as the computational models described here, can help to translate research findings obtained at a molecular or cellular level in different species into assessment of risk associated with alcohol exposure during human development.

“Daedalus, an architect famous for his skill, constructed the maze, confusing the usual marks of direction, and leading the eye of the beholder astray by devious paths winding in different directions. Thanks to the help of the princess Ariadne, Theseus rewound the thread he had laid, retraced his steps, and found the elusive gateway.” —Ovid, 17–18 A.D.

More than 30 years ago, fetal alcohol syndrome (FAS) was first described as a distinctive pattern of physiological and behavioral characteristics observed in children who had been exposed to alcohol in the womb (i.e., in utero) (Jones and Smith 1973; Lemoine et al. 1968). Since then, researchers have characterized a dose-response relationship between maternal alcohol consumption during pregnancy and a spectrum of disorders, ranging from more subtle neurodevelopmental effects termed alcohol-related neurodevelopmental disorder (ARND) at the lowest exposure levels (Sampson et al. 1997; Streissguth et al. 1980) to FAS at the highest exposure levels (Streissguth et al. 1980). Alcohol exposure in utero resulting in any one of these disorders has been estimated to affect nearly 1 in every 100 live births (Sampson et al. 1997), making it a serious public health concern.

Ever since the initial characterization of FAS, researchers have sought to understand potential mechanisms underlying alcohol’s toxic effects during development. Much of their research has focused on alcohol’s impact on brain development, and research advances in both normal and perturbed neurodevelopment have focused attention on the sensitive processes of neuronal generation, differentiation, and programmed cell death as critical in the etiology of neurodevelopmental disorders. However, the relative importance of these processes and their relationship to the dose and timing of alcohol exposure still are unknown. Systems biology approaches, such as genomic, transcriptomic, and proteomic analyses, may help fill this knowledge gap. Another strategy used in systems biology that may contribute to the elucidation of mechanisms contributing to alcohol-related neurodevelopmental deficits is the development of computational models that can incorporate data obtained using different experimental strategies from multiple species and from biological observations made at different levels of biological complexity (e.g., the molecular level versus the cellular level).

For the purposes of this article, which has the ultimate goal of relating disparate datasets in fetal alcohol research to projected outcomes in humans, the term “systems biology” is broadly defined. For this definition, the emphasis is on development of flexible computational tools for integration of data from multiple types of studies, including cell culture, rodent, and primate models across various end points such as behavioral-, organ-, cellular-, and molecular-level outcomes. This definition of systems biology builds on concepts presented in a report by the National Research Council (2000) calling for models that would integrate biological information from multiple levels of assessment to evaluate alcohol’s effects on development. The report emphasizes the importance of looking at alcohol’s impact at the molecular and cellular level and how these changes may impact the dynamics of development. The conceptual framework also includes the idea of incorporating information about the dose and duration of alcohol exposure (i.e., kinetic and exposure information) into this integrated model.

After reviewing some of the mechanisms that have been proposed to underlie ARND and FAS, this article introduces a computational model that applies data obtained in experiments assessing these potential mechanisms in order to quantitatively address their relative contributions. The article also discusses the relevance of these computational analyses to alcohol research and human health and points out additional areas of research that warrant investigators’ attention.

## Possible Mechanisms Underlying ARND

### Normal Development of the Mammalian Neocortex

The neocortex is the outermost layer of the mammalian brain and its most dominant structure. Although it is found in all mammals, its size is markedly increased in primates, particularly humans; thus, the surface area of the neocortex is 1,000 times greater in humans than in mice, even though the thickness of the neocortex increases only slightly (see Gohlke et al. 2007). In humans, the neocortex is the primary region involved in thought, language, and behavior. Studies in humans and animal models have demonstrated that the neocortex is particularly sensitive to alcohol-induced disturbances during development. Accordingly, many studies have focused on elucidating normal neocortical development and on determining alcohol’s effects on these processes. Most of these studies have been conducted in animal models, primarily mice and rats with a few primate studies, but information also is available from in vitro cell cultures and from studies on human neocortical development.

**Figure 1:**
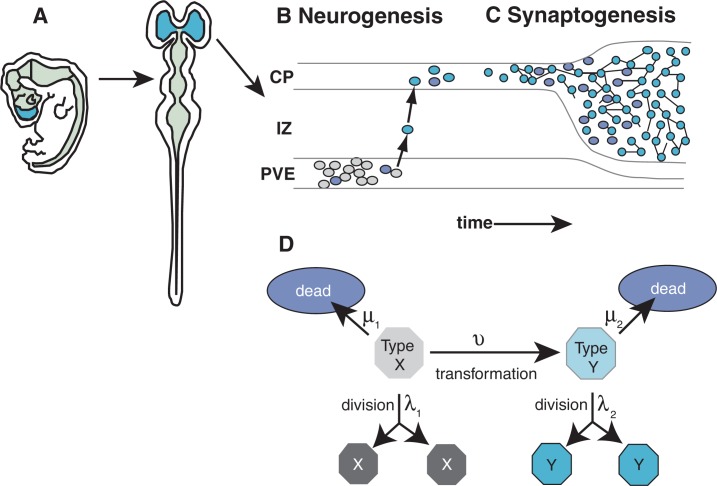
Mechanism-based framework for evaluating neocortical development. A) Illustration of the mammalian nervous system at the beginning of nerve cell development (i.e., neurogenesis) as seen from the side and back. Blue sections indicate the area where the neocortex will develop, green the rest of the central nervous system. B) During neurogenesis, progenitor cells for the neocortex are generated in the pseudostratified ventricular epithelium (PVE). Newly generated cells either die (purple cells), continue to proliferate (gray cells), or stop dividing and begin migrating through the intermediate zone (IZ) to the cortical plate (CP) (blue cells). C) In the CP, cells either differentiate into neurons that form synapses with neighboring cells (blue cells) or die by apoptosis (purple cells). D) Illustration of a basic model framework developed by Leroux and colleagues (1996) that was modified as a model for neocortical neurogenesis. Colors of cells indicate their place in the developing neocortex as illustrated in panels B and C. For example, type X cells represent neuronal progenitor cells in the PVE and type Y cells represent neurons leaving the PVE and migrating to the CP. Greek letters λ, μ, and ν represent the rates at which the respective cells divide, die, or are transformed, respectively. The model emphasizes that the transformation rate is dependent upon the division rate. SOURCE: Adapted from Gohlke et al. 2005.

Across all mammals, neocortical development follows the same pattern (see figure 1). The first phase is the generation of new nerve cells (i.e., neurogenesis). During this phase, which occurs on embryonic days 12 to 18 (E12 to E18) in rats and during the second trimester of pregnancy in humans, the cells that will eventually make up the neocortex are formed in a region of the embryo known as the pseudostratified ventricular epithelium (PVE).^1^ When the cells in this region divide, some of the daughter cells stay behind to undergo additional division. The others migrate away from the PVE, through a region called the intermediate zone, to their final location in the developing embryo known as the cortical plate. Once they have reached the cortical plate, the precursor cells differentiate into specialized nerve cells (i.e., neurons) and begin to establish multiple connections with neighboring neurons. This process is called synaptogenesis.^2^ During synaptogenesis, however, a substantial portion of the newly formed neurons fail to establish correct connections with other neurons and are therefore eliminated via programmed cell death (i.e., apoptosis). In rats, synaptogenesis and apoptosis occur after birth, on postnatal days 4 through 11 (PD4 through PD11); this corresponds to neurodevelopmental events occurring in the third trimester of pregnancy in humans.

### Alcohol’s Effects on Brain Development

Although the specific mechanisms underlying alcohol’s detrimental effects on the developing nervous system are not fully understood, researchers have used several strategies to investigate alcohol’s effects on brain development and, more specifically, on neurogenesis, synaptogenesis, and apoptosis. Some investigators have suggested that the inhibition of cellular growth and division (i.e., proliferation) during neurogenesis and enhanced induction of apoptosis during synaptogenesis may be particularly important in the development of ARND (Ikonomidou et al. 2000; Miller 1986). For example, various studies have shown that alcohol is a potent inhibitor of cellular proliferation, particularly in the developing brain (e.g., Laev et al. 1995; Pennington et al. 1984). Thus, alcohol may reduce the proportion of precursor cells that remain in the PVE to undergo further cell division or may increase the time it takes for the precursor cells to divide so that fewer cycles of cell division can occur during the time window available for neurogenesis (Guizzetti and Costa 1996; Miller 1989, 1992; Miller and Kuhn 1995).

To analyze neurogenesis in more detail, researchers have labeled DNA in developing mouse or rat embryos using compounds that can be measured easily. Such analyses found, for example, that alcohol exposure could reduce the number of cells in the PVE that are dividing and could increase the time it takes each cell to grow and divide into two daughter cells (i.e., the length of each cell cycle) (e.g., Miller and Kuhn 1995). Moreover, these effects were seen at or below blood alcohol concentrations (BACs) of 150 mg/dl in animals, which in humans is approximately twice the legal limit of 0.08 percent for driving.

Other studies have demonstrated alcohol’s ability to alter the natural waves of apoptosis during synaptogenesis (Ikonomidou et al. 2000; Climent et al. 2002). For each brain region, synaptogenesis occurs during a discrete period of time that coincides with a period of increased susceptibility to alcohol-induced neuronal death (Ikonomidou et al. 2000). The exact mechanisms through which alcohol induces apoptosis are unknown, but Ikonomidou and colleagues (2000) have suggested that alcohol’s effects on certain brain chemicals involved in the transmission of nerve signals (i.e., neurotransmitters) may trigger apoptosis during synaptogenesis in many brain regions.

Cell death during synaptogenesis can be studied by using selective stains that specifically label cells undergoing apoptosis or by analyzing the activity of certain enzymes (e.g., caspase 3) that are activated specifically during apoptosis. (As mentioned earlier, synaptogenesis occurs within about a week after birth in rodents but begins during the third trimester of pregnancy in humans.) Using a technique called DeOlmos silver staining, Ikonomidou and colleagues (2000) assessed the number of apoptotic cells in the neocortex of 8-day-old rats that were treated with alcohol, resulting in BACs of 500 mg/dl.^3^ The study found that the brain tissue of the alcohol-exposed animals contained 15 times more apoptotic neurons than the tissue of control animals. Additional experiments using different alcohol doses further demonstrated a dose-response relationship, suggesting that alcohol doses producing peak BACs of 200 mg/dl or more for more than 4 hours significantly increased the observed number of apoptotic neurons in the developing brain compared with control animals. The effect became progressively more severe the longer the BAC exceeded 200 mg/dl (Ikonomidou et al. 2000). Other experiments measuring caspase 3 activation as an indicator of apoptosis in 7-day-old mice found increased levels of apoptosis at even lower peak BACs of approximately 50 mg/dl when these levels were present for 30 to 45 minutes (Olney et al. 2002; Young and Olney 2006).

Finally, an approach to studying alcohol’s effects on overall brain development is to conduct studies of the three-dimensional characteristics of a cell or tissue (i.e., stereological analyses). These analyses are conducted with microscopic measurements that allow investigators to obtain a three-dimensional image of the tissue (e.g., brain region) under investigation and to determine, for example, the number of cells in that brain region. Several studies conducted in different brain regions of rats and mice found that, depending on the timing of exposure and BACs achieved, alcohol exposure could result in a reduction of cell numbers by up to one-third in the brain regions tested (see Gohlke et al. 2002).

Thus, all of these studies have demonstrated that alcohol exposure during development can lead to a reduction in cell numbers in the developing brain through several mechanisms. Because the different types of experiments were done in isolation and focused only on specific processes or developmental stages, however, they shed no light on the relative importance of these mechanisms and their potential interactions. In particular, the differential contributions of inhibition of proliferation and induction of cell death to ARND and FAS have yet to be elucidated. To obtain a more comprehensive picture of alcohol’s effects on the developing brain, systems biology approaches that can integrate diverse data would be useful. Such approaches could generate molecular, cellular, anatomical, and behavioral data using a wide variety of experimental designs as well as provide quantitative models that integrate these data (Andersen et al. 2005; Cummings and Kavlock 2005).

## A Systems-Based Computational Model for Studying ARND

One aspect of systems biology that can be introduced into the study of ARND is to develop computational models that can integrate data obtained using various experimental strategies. As a first step toward this goal, a general computational model for the developmental processes of proliferation, differentiation, and cell death has been applied to evaluate the potential mechanisms and impacts of a variety of environmental agents. The initial model was developed in 1996 and was previously applied to evaluate the neurodevelopmental impacts of methyl mercury (Faustman et al. 1999; Leroux et al. 1996). This model was subsequently extended to include neocortical development in mice, rats, monkeys, and humans, allowing for the assessment of alcohol’s effects on the developing brain and elucidating some of the mechanisms contributing to the pathogenesis of ARND (Gohlke et al. 2002, 2004, 2005, 2007). This approach is discussed in the following sections.

### Computational Models of Neurodevelopment

The researchers developed their computational models based on published quantitative experimental data (such as the data described in the previous section) describing cell cycle kinetics and cell death in the developing brains of mice, rats, monkeys, and humans. The models link effects at the cell level to effects at the organ level by simulating changes in neuron numbers in the adult based on the production and death of neurons in the developing organism. Furthermore, the models are expected to serve as a foundation for future application of data at the molecular and behavioral levels.

Construction of the models was based on the hypothesis that rapidly dividing, differentiating, and dying cells within a developing organ represent a sensitive target for environmental insults, such as alcohol exposure (Faustman et al. 1999; Leroux et al. 1996). This hypothesis is especially relevant for neurodevelopment, in which disruption occurring during the discrete periods of neurogenesis, migration, and synaptogenesis will result in specific malformations (see Gohlke et al. 2005). For example, disruption of neurogenesis most likely will result in overall reduction of cell number, manipulations interfering with cell migration likely will result in abnormal locations of neurons, and factors interrupting differentiation signals during synaptogenesis likely will result in apoptosis or abnormalities in the connections among neurons.

For their models, the investigators generated mathematical equations that track the fate of precursor cells as a function of division, differentiation, and death rates. Their models include division and death rates for precursor cells in the PVE as well as assessment of death rates for the differentiated neurons that have migrated to their final location and have begun the process of synaptogenesis. (The current models do not, however, incorporate the rate of migration of neurons from the PVE to the cortical plate.) The rate equations linking these processes are derived based on existing experimental data on normal neurodevelopment and on the impact of alcohol and other toxic substances during various developmental stages. To validate the model, the predictions derived from the application of these equations were compared with stereological data on cell numbers.

### Results Obtained With the Computational Model

**Figure 2:**
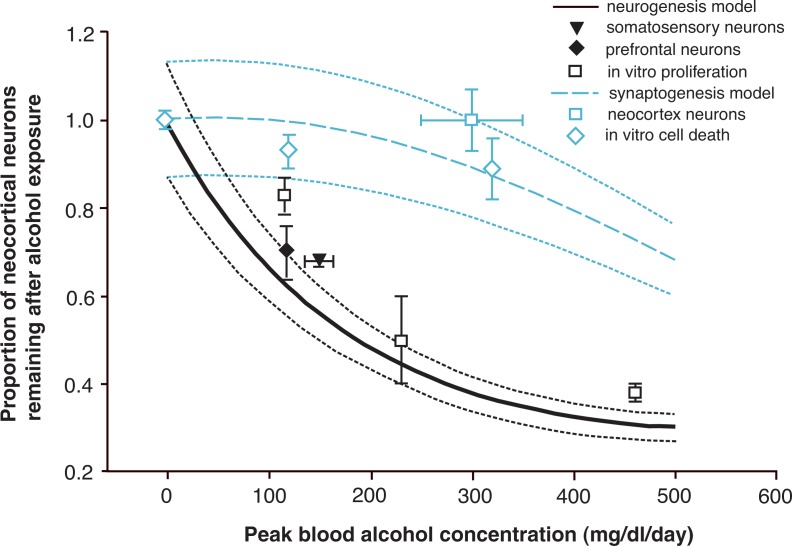
Neuronal loss in rats predicted by computer models of the effects of varying levels of alcohol exposure during the period of nerve cell formation (i.e., neurogenesis) versus the period of formation of connections with neighboring neurons (i.e., synaptogenesis). The solid black line represents the predicted neuronal loss resulting from alcohol-induced inhibition of neurogenesis, whereas the hatched blue line represents neuronal loss resulting from alcohol-induced induction of cell death during synaptogenesis. For comparison, experimental data of long-term neuronal loss in different regions of the neocortex (solid black triangle, solid black diamond and open blue square) determined by microscopic analysis of alcohol-exposed animals are shown, as are data obtained studying proliferation (open black squares) and cell death (open blue diamonds) of different types of brain cells grown in tissue culture (i.e., in vitro). These data show that alcohol exposure can interfere with both neurogenesis and synaptogenesis as predicted by the computer models. NOTE 1: The models are based on experimental data by Ikonomidou et al. 2000 for the synaptogenesis model and by Miller and Kuhn 1995 for the neurogenesis model. NOTE 2: Error bars represent standard errors reported for responses or ranges reported for peak blood alcohol concentrations. SOURCE: Gohlke et al. 2005.

The computational model was used to examine the role of reduced production of neuron precursors in the PVE in alcohol-induced impairments of brain development. The investigators examined how alcohol-induced inhibition of cell division of the precursor cells would affect the final number of neurons in the neocortex. Using the experimental data on cell cycle length and proportion of dividing cells obtained by Miller and Kuhn (1995), the model predicted that in rat embryos exposed to maternal BACs of about 150 mg/dl, the final number of neurons generated during neurogenesis would be about 30 percent lower than in control rat embryos (see figure 2) (Gohlke et al. 2005). This is consistent with the results of other studies demonstrating that cell numbers in the neocortex of rats prenatally exposed to alcohol were 33 to 35 percent lower than in control rats (Miller 1996; Miller and Potempa 1990). Thus, the predictions obtained with the computational model indicate that alcohol-induced changes in cell cycle length during early neurogenesis alone can account for the permanent cell loss observed in the neocortex of rats prenatally exposed to alcohol.

In a second step, the model was expanded to also evaluate alcohol-induced apoptosis during synaptogenesis (Gohlke et al. 2005). The model was based on data obtained by other investigators who had used either DeOlmos silver staining (Ikonomidou et al. 2000) or caspase 3 activation (Olney et al. 2002) to assess apoptosis. For both datasets, Gohlke and colleagues (2005) used the computational model to predict the relationship between increasing alcohol exposure and neuronal loss. These simulations demonstrated somewhat different dose-response relationships for the two datasets, with data based on silver staining analyses demonstrating greater cell loss in the neocortex than caspase 3 analyses. Moreover, significant neuronal loss in the neocortex was predicted only at the highest alcohol dose (i.e., peak BACs in the newborn rats of 500 mg/dl).

Finally, the investigators compared the model for alcohol-induced cell death during synaptogenesis with the model of alcohol-induced cell cycle prolongation during neurogenesis. This comparison found that the alcohol-induced lengthening of the cell cycle during neurogenesis results in greater deficits in neuron numbers in the adult than the alcohol-induced increase in cell death during synaptogenesis (Gohlke et al. 2005). For example, at peak BACs of 150 mg/dl, the neurogenesis model predicted a decrease of 35 to 40 percent in adult neuron numbers, whereas the synaptogenesis model predicted a decrease of 7 to 9 percent. These findings are consistent with effects of alcohol-induced cell death and alcohol-induced inhibition of cell proliferation in cultured neuronal cells (Miller 2003).

Thus, a system-based computational model allows researchers to directly link alcohol’s effects on specific cellular mechanisms to a final outcome on neuronal number in the adult and to estimate the potential relative contributions of these mechanisms across a range of alcohol doses and during various developmental stages. The current model addresses alcohol-induced decreases in cell numbers that could be extended to consider other potential alcohol-induced deficits, such as formation of insufficient or improper synapses between the existing neurons or alterations in the structure of the existing neurons. The neocortex is a relatively large brain region comprising several sub-regions that may not display a uniform development, and these regions may differ in their susceptibility to alcohol’s toxic effects during development. Therefore, additional experimental data are needed to fully model the effects of prenatal alcohol exposure on different regions within the neocortex.

## Relevance of Computational Models for Alcohol Research and Human Health

Computational models such as the one presented here offer the following benefits to researchers studying the effects of alcohol and other toxic substances during development and their impact on different organs:

- They provide a framework for assessing, organizing, and synthesizing research across multiple systems (e.g., the brain and other organs) and approaches (e.g., data obtained using different techniques).
- They provide approaches for translating research findings obtained from various species into information useful for predicting effects in humans.
- They allow researchers to determine the impact of available and missing data. This information can be useful in prioritizing subsequent experiments aimed at providing critical missing information.
- They provide a framework for testing hypotheses regarding the relative contributions of various modes of action of alcohol or other toxic substances (e.g., inhibition of cell proliferation vs. induction of cell death). Analyses of impacts on other brain regions are also possible by extending these frameworks.
- Because modeling approaches require a rigorous description of the processes involved, they also help identify those research areas in which information and knowledge still are lacking. As a result, the use of computational models can facilitate the formulation and testing of new hypotheses to elucidate the molecular mechanisms underlying alcohol-induced developmental disorders.

One important consideration in evaluating this and other models and determining their relevance to human health is that they often rely on data obtained in rodents. When extrapolating scientific findings from rodents to humans, however, it is crucial to acknowledge that the neocortex is much larger in humans than in other primates or mammals. Although the overall architecture of the brain is conserved in all mammals, the absolute and relative size of the neocortex varies greatly among species, with the neocortex occupying anywhere from 25 to 80 percent of the brain (Clark et al. 2001). Moreover, neocortex development is marked by discrete stages—such as progenitor cell proliferation and death, neuronal differentiation, and neuronal cell death during synaptogenesis— that differ among mammalian species with respect to their length and timing during gestation. Therefore, if researchers want to determine how specific perturbations during development may cause long-term neocortical deficits in humans, they must take into consideration the evolutionary changes in the cellular mechanisms underlying neocortical development in humans compared with those of commonly used model organisms, such as rodents. Systems-based models can facilitate this interpretation.

To address this issue, Gohlke and colleagues (2007) developed computational models of neocortical development in Rhesus monkeys and humans. These models are based on specific experimental studies in monkeys and humans that measure the duration of the neurogenesis period, cell cycle length, and proportion of cells labeled for death during neocortical development. When comparing these models with those for mice and rats, the investigators found that the previously determined differences between rodents and primates in the duration of the neurogenesis period and cell cycle length, as well as in the death rate during synaptogenesis, can account for the differences in neuronal cell numbers—and thus neocortical size—between rodents and primates. Moreover, the investigators predicted that death of developing neurons during synaptogenesis may play a greater role in shaping the adult brain in primates than in rodents. Such differences may, for example, help in determining the relative impact on brain development of alcohol-induced cell death during synaptogenesis. In a recent publication comparing model results with epidemiological as well as animal literature, the investigators suggest that the developing human neocortex may be more sensitive to the effects of alcohol than the developing rodent neocortex based on the relative increase in the length of neurogenesis and subsequent size of the neocortex in humans (Gohlke et al. 2008) Therefore, interspecies differences in the processes underlying neocortical development must be taken into consideration when extrapolating findings obtained in rodents.

## Lessons Learned

The existing rodent models of brain development and of alcohol’s impact on it already have provided researchers with important information and allow several conclusions regarding alcohol’s effects on brain development and the pathogenesis of ARND. For example, the current models described above suggest that alcohol-induced inhibition of precursor cell proliferation during neurogenesis may have a greater long-term impact on cell numbers in the neocortex than alcohol-related induction of apoptosis during synaptogenesis. In a next step, this information must be linked with a physiologically based model of the toxicokinetics of alcohol in the developing organism—that is, a model of how alcohol is taken up into the body, distributed throughout the blood and tissues, metabolized, and eliminated from the body—for an even more comprehensive systems approach to studying ARND. Such an approach may allow researchers to determine in more detail the neurotoxic effects of different alcohol doses at different developmental stages. This approach already has been used to model the effects of another neurotoxic substance, methylmercury (Lewandowski et al. 2002).

Such a systems approach can be particularly useful for determining the consequences of lower levels of prenatal alcohol exposure. As stated earlier, many of the existing rodent models discussed here have used nonphysiological alcohol concentrations. In contrast, there currently are minimal data on the effects of alcohol doses resulting in BACs less than 100 mg/dl. Experimental evidence from Rhesus monkeys suggests, however, that neurogenesis may be affected even by relatively low levels of alcohol exposure (i.e., peak BACs of approximately 20 mg/dl) early during gestation (Schneider et al. 2001). Therefore, studies should be conducted to model the effects of lower levels of alcohol exposure.

### Need for Additional Data on Neocortical Development

The computational models discussed here have been used to assess the contributions of two potential modes of action—inhibition of proliferation and induction of cell death—through which alcohol may interfere with normal neocortical development. Although these analyses provide important information on the relationship between the dose of alcohol to which a developing fetus is exposed and the resulting effects on the neocortex, they also underscore that it is essential to determine the sensitivity of the developing brain to alcohol and other neurotoxic substances during discrete developmental stages. In other words, to fully understand the risk associated with prenatal alcohol exposure it is important to analyze not only what alcohol concentrations the developing fetus is exposed to but also when the exposure occurs because each organ or tissue may be particularly susceptible to alcohol’s effects during specific developmental stages. For example, although alcohol exposure during synaptogenesis induced a substantial increase in apoptotic neurons, this transient response did not appear to confer a significant long-term neuronal loss at lower levels of exposure. Conversely, relatively small and potentially harder-to-detect lengthening of the cell cycle at the beginning of neurogenesis was predicted to result in massive neuronal deficits in the mature neocortex. For developing accurate models of the long-term impact of a transient exposure to alcohol or other neurotoxic substances, it is therefore important to understand the context of the underlying developmental processes during a particular exposure period.

The extensive data found in the literature regarding alcohol’s developmental neurotoxicity are an excellent source for evaluating such a computational modeling approach. Although alcohol can have numerous neurotoxic effects at the molecular and cellular level, depending on the dose and time of exposure (for a review, see Maier and West 2001), a modeling approach focusing on one or a few modes of action (e.g., reduction in cell number) may be sufficient to describe alcohol’s key toxic effects for public health risk assessment purposes. Such a “mode of action” modeling methodology has the potential to vastly improve the usage of scientific data for assessing the risk associated with exposure to toxic substances during development because it provides a quantitative framework in which cellular and eventually molecular effects can be linked to an adverse neurodevelopmental outcome, such as ARND. Detailed molecular studies have suggested a variety of changes that could explain the alterations in cell cycle progression, cell migration, and apoptosis described in the integrated models presented here (see, for example, Lee et al. 2004; Green et al. 2007; Joshi et al. 2006; Peng et al. 2004). The expansion of these models to include more detailed mechanistic hypotheses, such as effects on cell adhesion and motility (Wilkemeyer et al. 2000) or genomics datasets (Green et al. 2007), will allow for further quantitative mechanistic comparisons, informing basic science research and development of potential pharmacological interventions.

In summary, systems-based approaches, such as the computational models described here, can help to translate research findings obtained at a molecular or cellular level to actual assessment of risk associated with alcohol exposure during human development. Moreover, the generation of such models may help establish a two-way dialogue whereby modeling efforts help identify critical research needs and new data are incorporated into the framework of an existing model to determine their relevance and impact, increasing researchers’ ability to translate research generated across species for estimating human risks and determining clinical relevancy.

## Footnotes

1 In addition to the PVE, two other brain regions called the subventricular zone and the medial ganglionic eminence also contribute cells to the neocortex. However, the cells from these regions were not included in the computational models discussed later in this article because their contributions to the neocortex are relatively minor.

2 Synapses are the regions where neurons establish contacts to neighboring neurons and where nerve signals are transmitted from one neuron to another. Each neuron typically interacts with many other neurons.

3 Note that a BAC of 500 mg/dl (i.e., 0.5 percent) is not normally found in humans, and even a BAC of 200 mg/dl (i.e., 0.2 percent) as mentioned in the following sentences is not commonly found and is associated with severe intoxication in humans.
